# Widespread *gpa-4* promoter-driven expression during *Caenorhabditis elegans* development

**DOI:** 10.17912/micropub.biology.000443

**Published:** 2021-08-19

**Authors:** Jaime Osuna-Luque, Collin Y Ewald, Peter Meister

**Affiliations:** 1 Institute of Cell Biology, University of Bern; 2 ETH Zürich

## Abstract

The *gpa-4* promoter-driven expression is described as specific for ASIL and ASIR chemosensory neurons in the nematode *Caenorhabditis elegans*, yet this was mostly examined in adult animals. Here we used a recombination-mediated reporter to test the previously used *gpa-4 *promoter expression. This reporter highlights all cells in which the *gpa-4 *promoter has been active at one point or another during development. We show that the *gpa-4 *promoter is indeed active in ASI, yet to our surprise, this**promoter drives also expression in many other cell types, including the somatic gonad, the seam cells, a subset of tail and head neurons, and muscle cells, demonstrating a widespread activity of this transgenic *gpa-4* promoter during embryonic and post-embryonic development.

**Figure 1. gpa-4 promoter expression during development assessed using recombination-mediated red-to-green fluorescent reporter:**
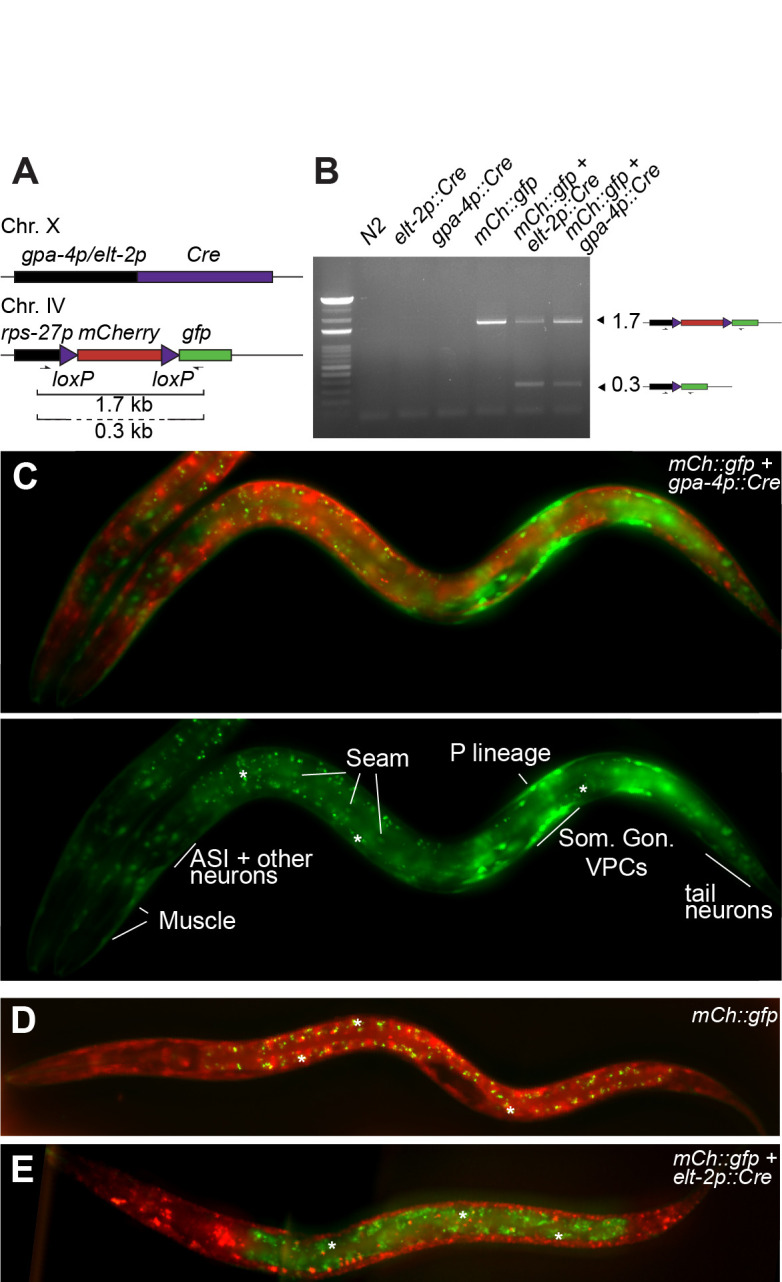
(A) Experimental system to assess the expression pattern of the *gpa-4* promoter during development. Expression of the Cre recombinase driven by the *gpa-4* promoter leads to the excision of a floxed mCherry cassette and the expression of GFP. Any cell type in which the *gpa-4* promoter has been active will thereby appear green (B) PCR across the floxed mCherry cassette using primers depicted in A, in the wild-type strain (N2), a strain containing only the floxed *mCherry::GFP* transgene and a strain carrying both the floxed *mCherry::GFP* cassette and the *gpa-4p::Cre* construct. Band sizes in kb. (C) Many lineages express GFP, following excision of the floxed *mCherry* cassette by Cre driven by the *gpa-4* promoter during embryonic and post-embryonic development. Fluorescent compound image of an L2 larval stage animal with red (unexcised cassette) and green (excised cassette) cells. Clear expression is identified in the somatic gonad (Som. Gon.), vulval precursor cells (VPC), seam cells, body wall muscle cells, tail, and head neurons, as well as, the P lineage. *: autofluorescence of gut granules (D) Negative control animal showing no GFP expression, as they carry only the floxed *mCherry* cassette transgene without a Cre driver (E) Positive control animal in which Cre is active in the intestine (*elt-2* promoter): intestine-specific excision of the *mCherry* cassette leads to GFP expression in the gut.

## Description

In *C. elegans,* the *gpa-4* gene encodes a G-protein α-subunit, part of an evolutionary conserved Gα family expressed in muscle and neuronal cells (Jansen *et al.* 1999). Previous studies using fluorescent reporters have suggested that the *gpa-4* promoter drives expression exclusively in the head chemosensory neurons ASIL and ASIR (Jansen *et al.* 1999). This led subsequent studies to consider the *gpa-4* promoter as ASI-specific (see Table 1 for all studies using the *gpa-4* promoter to drive gene expression).

Recently, red-to-green fluorescent reporter switches have been developed to validate promoter-driven transcriptional patterns and tissue specificity. These systems use either *Cre/lox* or FLP/FRT recombination. Their mode-of-operation, in principle, is identical and relies on two different transgenes. First, the recombinase is placed under transcriptional control of the promoter to be tested. Second, a ubiquitous promoter drives the expression of a red fluorophore, which is excised by the recombinase leading to the expression of a green fluorescent protein (Ruijtenberg and van den Heuvel 2015; Muñoz-Jiménez *et al.* 2017). As the red-to-green switch is irreversible, any cell or lineage in which the promoter has been active during development will be labeled green, while the rest of the animal will be red.

To examine the expression pattern of the *gpa-4* promoter, we generated a *gpa-4p::Cre* transgene integrated on Chr X using MosSCI (Frøkjær-Jensen *et al.* 2012; Figure 1A). Recombination efficiency was first tested by PCR with primers encompassing the floxed mCherry cassette (Figure 1A,B). In the absence of recombination (*mCherry::gfp* cassette), a single product of 1.7 kb was amplified. When recombination occurs, two products will be visible, as most cells in the animal have the intact *mCherry::gfp* cassette while in the target tissue of the Cre driver construct, the mCherry cassette is deleted. This is indeed the case for both the positive control intestinal Cre driver (*elt-2p::Cre*) and the *gpa-4p::Cre* one, demonstrating that Cre is expressed and active when expressed under transcriptional control of the *gpa-4* promoter. The intensity of the lower, recombined band was, however, surprisingly intense for an event which should only happen in the two ASI neurons per animal. Indeed, microscopic examination of animals showed clear and reproducible expression of GFP in many tissues, such as the somatic gonad, vulval precursor cells, seam cells, body wall muscle cells, tail and head neurons, as well as, the P lineage (Figure 1C). Both negative and positive controls showed the expected fluorescence pattern (no GFP and GFP expression only in the intestine, respectively; Figure 1D,E). Recombination by Cre driven by the *gpa-4* promoter occurs therefore in many more cells than the published *gpa-4* transcriptional fusion (Jansen *et al.* 1999) in which ASI-specific expression was reported.

One limitation of our approach could be that the insertion site of the *gpa-4p::Cre* influences its expression. However, there are two reasons arguing against this possibility. First, the same single copy insertion site was used for a variety of *Cre* drivers (muscle, intestine, mesoblast, vulva and XXX neuroendocrine cells; Ruijtenberg and van den Heuvel 2015; Gómez-Saldivar *et al.* 2020). Expression patterns for the different promoters did in each case correspond to the expected expression pattern. Second, single cell RNA-seq in L1 larvae (Cao *et al.* 2017) detected the endogenous *gpa-4* transcript in cells identified as somatic gonad precursors, vulval precursors, sex myoblasts and ciliated sensory neurons (Extended data). These tissues overlap with the cell types in which we identified *gpa-4* promoter activity and Cre expression. Taken together, we conclude that the previously thought ASI-specific transgenic *gpa-4* promoter is widely active during embryonic and larval development.

## Methods

**Worm growth**

Worms were grown on solid nematode growth media (NGM), seeded with OP50 bacteria for maintenance culture and genetic crosses. All animals were grown at 22°C. Male animals were induced at 30ºC for 5 hours to carry out genetic crosses. For microscopy experiments using the iMIC platform, synchronized L1s were seeded onto 45 mm plates. Microscopy experiments were carried out after 12 hours.

**Fluorescence imaging**

Animals were laid on a pad of 2% agarose in water supplemented with 0.01% sodium azide, on a microscope slide, covered with a drop of M9 buffer and a coverslip. Fluorescence imaging was carried out on an iMIC widefield microscope (FEI) with a 40x oil objective and an Hamamatsu Orca camera. Z stacks were acquired with a slice separation of 1 μm and deconvolved using SVI Huygens before maximal intensity projection along the z-axis.

**Cloning of *gpa-4 Cre* driver**

The plasmid used in this study was generated using Gibson assembly. The *gpa-4* promoter was amplified from plasmid pLSD146, in which it drives expression of GFP (Jansen *et al.* 1999) using primers 1581/1582. The degron sequence was amplified from plasmid #503 using primers 1583/1584. The rest of the plasmid which contains the *Cre::tbb-2* 3’UTR sequence and the backbone pCFJ355 (Frøkjær-Jensen *et al.* 2012), including homology regions to the ttTi14024 *Mos* insertion were amplified separately in two pieces using primers 1577/1578 and 1579/1580 and the plasmid pSR33 as a template (Ruijtenberg and van den Heuvel 2015). All PCR reactions were then digested with *Dpn*I and fragments ligated together using Gibson assembly. The Cre ORF ATG in the new plasmid is located at the same place relative to the *gpa-4* promoter than in the original pLSD146 plasmid.

## Reagents

**Primer collection.**

**Table:** 

**Primer name**	**Sequence**	**Template**
PM1581	*GAGGGTACCAGAGCTCACCTAGGTATCTAGCAAGCTTGCGACTTTCGATACGTAGGTCTC*	pLSD146
PM1582	*CTTATTCATTTTGTGAACACTTTTCAACAAAGCTTGATGCCTAAAGATCCAGCCAAACCTCCGGCC*	pLSD146
PM1583	*CATTTTGTGAACACTTTTCAACAAAGCTTGATGCCTAAAGATCCAGCCAAACCTCCGGCC*	#503
PM1584	*GTAGTCTCCATCGTGATCCTTGTAATCCATCTTCACGAACGCCGCCGCCTCCGGGCC*	#503
PM1577	*ATGGATTACAAGGATCACGATGGAGACTACAAGG*	#328
PM1578	*CCCTGGCGTTACCCAACTTAATCGCCTTGC*	pSR33
PM1579	*GCAAGGCGATTAAGTTGGGTAACGCCAGGG*	pSR33
PM1580	*GCTAGATACCTAGGTGAGCTCTGGTACCCTCTAG*	pSR33
PM1323	*CCTCGTTTCGAAGTTGGTTTG*	gDNA
PM1324	*CTCCGGTGAAGAGCTCCTCTCC*	gDNA

**Plasmids**

**Table:** 

**Plasmid name**	**Source**	**Plasmid information**	**Backbone**
#522	This study	*gpa-4p::Degron::Cre::tbb-2 3’UTR*	pCFJ355
pLSD146	Jansen *et al.*1999	*gpa-4p::gfp*	–
#503	Gomez, Osuna-Luque *et al.*, 2020	*hsp16.2p::lox::mCherry::lox::degron::Dam::rpb-6::unc54 3’UTR*	pCFJ151
pSR33	Ruijtenberg and van der Heuvel, 2015	*hsp16.2p::Cre::tbb-2 3’UTR*	pCFJ355

***C. elegans* strains**

Plasmid #522 was integrated as a single copy using MosSCI on the chromosome X *ttTi14024 Mos* insertion site (Frøkjaer-Jensen *et al.* 2008).

**Table:** 

**Strain name**	**Source**	**Genotype**
N2	CGC	*Wild type Bristol N2*
PMW848	This study	*ubsSi45[gpa-4p::degron::Cre::tbb-2 3’UTR; unc-119(+) @ttTi14024] X*
SV1361	Ruijtenberg and van der Heuvel, 2015, CGC	*heIs105 [rps-27::loxP::nls::mCherry::let858 3’UTR::loxP::nls::GFP::let-858 3’UTR; unc-119(+) @cxTi10816] IV*
SV1439	Ruijtenberg and van der Heuvel, 2015, CGC	*heSi142[elt-2p::egl-13nls::Cre::tbb-2 3’UTR; unc-119(+) @ttTi14024] X*
PMW417	This study	*heSi104[rps-27p::loxP::nls::mCh::let-858 3’UTR::loxP::nls::gfp::let-858 3’UTR;unc-119(+) @cxTi10816] IV; unc-119(?) III; heSi142[elt-2p::egl-13nls::Cre::tbb-2 3’UTR @ttTi14042] X*
PMW867	This study	*heIs105 [rps-27::loxP::nls::mCherry::let858 3’UTR::loxP::nls::GFP::let-858 3’UTR; unc-119(+) @cxTi10816] IV; ubsSi45[gpa-4p::degron::Cre::tbb-2 3’UTR; unc-119(+) @ttTi14024] X*

**Table 1. *gpa-4* promoter-driven gene expression studies**

**Table:** 

**First author**	**PMID**	***gpa-4* promoter driving expression of**
Gallagher *et al.*	23739968	*daf-11*, *egl-4*, *GCaMP2.2b*
Guo *et al.*	30428355	*R-GECO1 (red protein), TRPV1, TeTx, ser-3*
Cheong-Cheong *et al.*	25898004	*npr-17::SL2::RFP*
Broekhuis *et al.*	23444385	*sql-1::GFP, aman-2::YFP*
Murakami *et al.*	11677050	*daf-11 cDNA*
Chen *et al.*	27585848	*NLS::mCherry,* mouse OxR2
Pandey *et al.*	33712439	*exp-1::SL2::wrmScarlet, str-2::SL2::wrmScarlet*
Kim *et al.*	16143323	*unc-3, GFP*
Dixit *et al.*	32432390	*str-2*
You *et al.*	18316030	*daf-7*
Bishop *et al.*	17538612	*skn-1b::gfp*

## References

[R1] Cao J, Packer JS, Ramani V, Cusanovich DA, Huynh C, Daza R, Qiu X, Lee C, Furlan SN, Steemers FJ, Adey A, Waterston RH, Trapnell C, Shendure J (2017). Comprehensive single-cell transcriptional profiling of a multicellular organism.. Science.

[R2] Frøkjaer-Jensen C, Davis MW, Hopkins CE, Newman BJ, Thummel JM, Olesen SP, Grunnet M, Jorgensen EM (2008). Single-copy insertion of transgenes in Caenorhabditis elegans.. Nat Genet.

[R3] Frøkjær-Jensen C, Davis MW, Ailion M, Jorgensen EM (2012). Improved Mos1-mediated transgenesis in C. elegans.. Nat Methods.

[R4] Gómez-Saldivar G, Osuna-Luque J, Semple JI, Glauser DA, Jarriault S, Meister P (2020). Tissue-Specific Transcription Footprinting Using RNA PoI DamID (RAPID) in *Caenorhabditis elegans*.. Genetics.

[R5] Jansen G, Thijssen KL, Werner P, van der Horst M, Hazendonk E, Plasterk RH (1999). The complete family of genes encoding G proteins of Caenorhabditis elegans.. Nat Genet.

[R6] Muñoz-Jiménez C, Ayuso C, Dobrzynska A, Torres-Mendéz A, Ruiz PC, Askjaer P (2017). An Efficient FLP-Based Toolkit for Spatiotemporal Control of Gene Expression in *Caenorhabditis elegans*.. Genetics.

[R7] Ruijtenberg S, van den Heuvel S (2015). G1/S Inhibitors and the SWI/SNF Complex Control Cell-Cycle Exit during Muscle Differentiation.. Cell.

